# Supplementary material for: Different detection capabilities by mycological media for Candida isolates from mono- or dual-species cultures
Source: PLoS One. 2020 Mar 23;15(3):e0226467. doi: 10.1371/journal.pone.0226467 (PMC7089522; doi:10.1371/journal.pone.0226467)
Supplement: S1 Table — (DOC) [file pone.0226467.s001.doc]

**S1 Table**

|  |  | **N. of detections obtained by all readers at the indicated times according to the culture medium** | | | | | | | | | | | |
| --- | --- | --- | --- | --- | --- | --- | --- | --- | --- | --- | --- | --- | --- |
| **Cultured *Candida* species** | | **BCG** | | | | **CHROM** | | | | **SDA** | | | |
|  |  | **24 h** | **48 h** | **72 h** | **All times** | **24 h** | **48 h** | **72 h** | **All times** | **24 h** | **48 h** | **72 h** | **All times** |
| *C. albicans* | – | 3 (1/1/1) | 3 (1/1/1) | 3 (1/1/1) | 9 (3/3/3) | 0 (0/0/0) | 3 (1/1/1) | 3 (1/1/1) | 6 (2/2/2) | 3 (1/1/1) | 4 (1/2/1) | 4 (1/2/1) | 11 (3/5/3) |
| *C. albicans* | *C. auris* | 6 (2/2/2) | 6 (2/2/2) | 6 (2/2/2) | 18 (6/6/6) | 5 (1/2/2) | 5 (1/2/2) | 5 (1/2/2) | 15 (3/6/6) | 4 (1/2/1) | 4 (1/2/1) | 4 (1/2/1) | 12 (3/6/3) |
| *C. albicans* | *C. dubliniensis* | 6 (2/2/2) | 6 (2/2/2) | 6 (2/2/2) | 18 (6/6/6) | 3 (1/1/1) | 6 (2/2/2) | 6 (2/2/2) | 15 (5/5/5) | 6 (2/2/2) | 6 (2/2/2) | 6 (2/2/2) | 18 (6/6/6) |
| *C. albicans* | *C. glabrata* | 6 (1/3/2) | 5 (1/2/2) | 5 (1/2/2) | 16 (3/7/6) | 3 (1/1/1) | 6 (2/2/2) | 6 (2/2/2) | 15 (5/5/5) | 5 (1/2/2) | 6 (2/2/2) | 6 (2/2/2) | 17 (5/6/6) |
| *C. albicans* | *C. guilliermondii* | 4 (1/2/1) | 6 (2/2/2) | 6 (2/2/2) | 16 (5/6/5) | 3 (1/1/1) | 6 (2/2/2) | 6 (2/2/2) | 15 (5/5/5) | 4 (1/2/1) | 4 (1/2/1) | 4 (1/2/1) | 12 (3/6/3) |
| *C. albicans* | *C. incospicua* | 5 (1/2/2) | 6 (2/2/2) | 6 (2/2/2) | 17 (5/6/6) | 4 (1/2/1) | 5 (1/2/2) | 5 (1/2/2) | 14 (3/6/5) | 6 (2/2/2) | 6 (2/2/2) | 6 (2/2/2) | 18 (6/6/6) |
| *C. albicans* | *C. kefyr* | 6 (2/2/2) | 6 (2/2/2) | 6 (2/2/2) | 18 (6/6/6) | 3 (1/1/1) | 6 (2/2/2) | 6 (2/2/2) | 15 (5/5/5) | 6 (2/2/2) | 5 (1/2/2) | 5 (1/2/2) | 16 (4/6/6) |
| *C. albicans* | *C. krusei* | 6 (2/2/2) | 6 (2/2/2) | 6 (2/2/2) | 18 (6/6/6) | 3 (1/1/1) | 6 (2/2/2) | 6 (2/2/2) | 15 (5/5/5) | 6 (2/2/2) | 6 (2/2/2) | 6 (2/2/2) | 18 (6/6/6) |
| *C. albicans* | *C. lusitaniae* | 5 (1/2/2) | 6 (2/2/2) | 5 (2/1/2) | 16 (5/5/6) | 4 (1/2/1) | 6 (2/2/2) | 6 (2/2/2) | 16 (5/6/5) | 6 (2/2/2) | 5 (1/2/2) | 5 (1/2/2) | 16 (4/6/6) |
| *C. albicans* | *C. nivariensis* | 4 (1/2/1) | 5 (1/2/2) | 5 (1/2/2) | 14 (3/6/5) | 3 (1/1/1) | 6 (2/2/2) | 6 (2/2/2) | 15 (5/5/5) | 5 (1/2/2) | 6 (2/2/2) | 6 (2/2/2) | 17 (5/6/6) |
| *C. albicans* | *C. norvegensis* | 6 (2/2/2) | 6 (2/2/2) | 6 (2/2/2) | 18 (6/6/6) | 4 (1/1/2) | 6 (2/2/2) | 6 (2/2/2) | 16 (5/5/6) | 6 (2/2/2) | 6 (2/2/2) | 6 (2/2/2) | 18 (6/6/6) |
| *C. albicans* | *C. parapsilosis* | 6 (2/2/2) | 6 (2/2/2) | 6 (2/2/2) | 18 (6/6/6) | 3 (1/1/1) | 3 (1/1/1) | 3 (1/1/1) | 9 (3/3/3) | 6 (2/2/2) | 6 (2/2/2) | 6 (2/2/2) | 18 (6/6/6) |
| *C. albicans* | *C. pararugosa* | 5 (2/2/1) | 6 (2/2/2) | 6 (2/2/2) | 17 (6/6/5) | 0 (0/0/0) | 6 (2/2/2) | 6 (2/2/2) | 12 (4/4/4) | 4 (2/1/1) | 6 (2/2/2) | 6 (2/2/2) | 16 (6/6/4) |
| *C. albicans* | *C. pelliculosa* | 6 (2/2/2) | 6 (2/2/2) | 6 (2/2/2) | 18 (6/6/6) | 3 (1/1/1) | 6 (2/2/2) | 5 (2/1/2) | 14 (5/4/5) | 5 (2/2/1) | 6 (2/2/2) | 6 (2/2/2) | 17 (6/6/5) |
| *C. albicans* | *C. robusta* | 6 (2/2/2) | 6 (2/2/2) | 6 (2/2/2) | 18 (6/6/6) | 3 (1/1/1) | 6 (2/2/2) | 6 (2/2/2) | 15 (5/5/5) | 4 (1/2/1) | 5 (1/2/2) | 5 (1/2/2) | 14 (3/6/5) |
| *C. albicans* | *C. sorbosa* | 7 (2/3/2) | 6 (2/2/2) | 6 (2/2/2) | 19 (6/7/6) | 3 (1/1/1) | 6 (2/2/2) | 6 (2/2/2) | 15 (5/5/5) | 5 (2/2/1) | 6 (2/2/2) | 6 (2/2/2) | 17 (6/6/5) |
| *C. albicans* | *C. tropicalis* | 6 (2/2/2) | 6 (2/2/2) | 6 (2/2/2) | 18 (6/6/6) | 3 (1/1/1) | 6 (2/2/2) | 6 (2/2/2) | 15 (5/5/5) | 6 (2/2/2) | 6 (2/2/2) | 6 (2/2/2) | 18 (6/6/6) |
| *C. albicans* | *C. utilis* | 6 (2/2/2) | 6 (2/2/2) | 6 (2/2/2) | 18 (6/6/6) | 4 (1/2/1) | 6 (2/2/2) | 6 (2/2/2) | 16 (5/6/5) | 6 (2/2/2) | 6 (2/2/2) | 6 (2/2/2) | 18 (6/6/6) |
| *C. auris* | – | 3 (1/1/1) | 3 (1/1/1) | 3 (1/1/1) | 9 (3/3/3) | 3 (1/1/1) | 3 (1/1/1) | 3 (1/1/1) | 9 (3/3/3) | 3 (1/1/1) | 3 (1/1/1) | 3 (1/1/1) | 9 (3/3/3) |
| *C. auris* | *C. dubliniensis* | 6 (2/2/2) | 6 (2/2/2) | 6 (2/2/2) | 18 (6/6/6) | 6 (2/2/2) | 6 (2/2/2) | 6 (2/2/2) | 18 (6/6/6) | 5 (1/2/2) | 5 (1/2/2) | 5 (1/2/2) | 15 (3/6/6) |
| *C. auris* | *C. glabrata* | 5 (1/2/2) | 5 (1/2/2) | 5 (1/2/2) | 15 (3/6/6) | 5 (1/2/2) | 5 (1/2/2) | 5 (1/2/2) | 15 (3/6/6) | 5 (1/2/2) | 5 (1/2/2) | 4 (1/1/2) | 14 (3/5/6) |
| *C. auris* | *C. guilliermondii* | 6 (2/2/2) | 6 (2/2/2) | 6 (2/2/2) | 18 (6/6/6) | 6 (2/2/2) | 6 (2/2/2) | 6 (2/2/2) | 18 (6/6/6) | 3 (1/1/1) | 3 (1/1/1) | 3 (1/1/1) | 9 (3/3/3) |
| *C. auris* | *C. incospicua* | 5 (1/2/2) | 6 (2/2/2) | 6 (2/2/2) | 17 (5/6/6) | 6 (2/2/2) | 6 (2/2/2) | 6 (2/2/2) | 18 (6/6/6) | 5 (1/2/2) | 5 (1/2/2) | 5 (1/2/2) | 15 (3/6/6) |
| *C. auris* | *C. kefyr* | 6 (2/2/2) | 6 (2/2/2) | 6 (2/2/2) | 18 (6/6/6) | 5 (1/2/2) | 5 (1/2/2) | 5 (1/2/2) | 15 (3/6/6) | 5 (1/2/2) | 5 (1/2/2) | 5 (1/2/2) | 15 (3/6/6) |
| *C. auris* | *C. krusei* | 5 (2/1/2) | 5 (2/1/2) | 5 (2/1/2) | 15 (6/3/6) | 6 (2/2/2) | 6 (2/2/2) | 6 (2/2/2) | 18 (6/6/6) | 6 (2/2/2) | 6 (2/2/2) | 6 (2/2/2) | 18 (6/6/6) |
| *C. auris* | *C. lusitaniae* | 6 (2/2/2) | 6 (2/2/2) | 6 (2/2/2) | 18 (6/6/6) | 5 (2/2/1) | 6 (2/2/2) | 6 (2/2/2) | 17 (6/6/5) | 3 (1/1/1) | 3 (1/1/1) | 3 (1/1/1) | 9 (3/3/3) |
| *C. auris* | *C. nivariensis* | 6 (2/2/2) | 6 (2/2/2) | 6 (2/2/2) | 18 (6/6/6) | 5 (1/2/2) | 5 (1/2/2) | 5 (1/2/2) | 15 (3/6/6) | 5 (1/2/2) | 5 (1/2/2) | 5 (1/2/2) | 15 (3/6/6) |
| *C. auris* | *C. norvegensis* | 6 (2/2/2) | 6 (2/2/2) | 6 (2/2/2) | 18 (6/6/6) | 5 (1/2/2) | 5 (1/2/2) | 5 (1/2/2) | 15 (3/6/6) | 6 (2/2/2) | 6 (2/2/2) | 6 (2/2/2) | 18 (6/6/6) |
| *C. auris* | *C. parapsilosis* | 6 (2/2/2) | 6 (2/2/2) | 6 (2/2/2) | 18 (6/6/6) | 6 (2/2/2) | 6 (2/2/2) | 6 (2/2/2) | 18 (6/6/6) | 6 (2/2/2) | 6 (2/2/2) | 6 (2/2/2) | 18 (6/6/6) |
| *C. auris* | *C. pararugosa* | 6 (2/2/2) | 6 (2/2/2) | 6 (2/2/2) | 18 (6/6/6) | 4 (1/2/1) | 4 (1/2/1) | 4 (1/2/1) | 12 (3/6/3) | 6 (2/2/2) | 6 (2/2/2) | 6 (2/2/2) | 18 (6/6/6) |
| *C. auris* | *C. pelliculosa* | 5 (1/2/2) | 6 (2/2/2) | 6 (2/2/2) | 17 (5/6/6) | 5 (1/2/2) | 5 (1/2/2) | 5 (1/2/2) | 15 (3/6/6) | 6 (2/2/2) | 6 (2/2/2) | 6 (2/2/2) | 18 (6/6/6) |
| *C. auris* | *C. robusta* | 6 (2/2/2) | 6 (2/2/2) | 6 (2/2/2) | 18 (6/6/6) | 6 (2/2/2) | 6 (2/2/2) | 6 (2/2/2) | 18 (6/6/6) | 5 (1/2/2) | 5 (1/2/2) | 5 (1/2/2) | 15 (3/6/6) |
| *C. auris* | *C. sorbosa* | 6 (2/2/2) | 6 (2/2/2) | 6 (2/2/2) | 18 (6/6/6) | 5 (1/2/2) | 5 (1/2/2) | 5 (1/2/2) | 15 (3/6/6) | 6 (2/2/2) | 6 (2/2/2) | 6 (2/2/2) | 18 (6/6/6) |
| *C. auris* | *C. tropicalis* | 6 (2/2/2) | 6 (2/2/2) | 6 (2/2/2) | 18 (6/6/6) | 6 (2/2/2) | 6 (2/2/2) | 6 (2/2/2) | 18 (6/6/6) | 6 (2/2/2) | 6 (2/2/2) | 6 (2/2/2) | 18 (6/6/6) |
| *C. auris* | *C. utilis* | 4 (1/1/2) | 4 (1/1/2) | 4 (1/1/2) | 12 (3/3/6) | 5 (1/2/2) | 5 (1/2/2) | 5 (1/2/2) | 15 (3/6/6) | 6 (2/2/2) | 6 (2/2/2) | 6 (2/2/2) | 18 (6/6/6) |
| *C. dubliniensis* | – | 3 (1/1/1) | 3 (1/1/1) | 3 (1/1/1) | 9 (3/3/3) | 4 (1/2/1) | 3 (1/1/1) | 3 (1/1/1) | 10 (3/4/3) | 3 (1/1/1) | 3 (1/1/1) | 3 (1/1/1) | 9 (3/3/3) |
| *C. dubliniensis* | *C. glabrata* | 8 (2/3/3) | 7 (2/2/3) | 7 (2/2/3) | 22 (6/7/9) | 6 (2/2/2) | 6 (2/2/2) | 6 (2/2/2) | 18 (6/6/6) | 5 (1/2/2) | 6 (2/2/2) | 6 (2/2/2) | 17 (5/6/6) |
| *C. dubliniensis* | *C. guilliermondii* | 6 (2/2/2) | 6 (2/2/2) | 6 (2/2/2) | 18 (6/6/6) | 6 (2/2/2) | 6 (2/2/2) | 6 (2/2/2) | 18 (6/6/6) | 6 (2/2/2) | 6 (2/2/2) | 6 (2/2/2) | 18 (6/6/6) |
| *C. dubliniensis* | *C. incospicua* | 6 (2/2/2) | 6 (2/2/2) | 6 (2/2/2) | 18 (6/6/6) | 3 (1/2/–) | 5 (1/2/2) | 5 (1/2/2) | 13 (3/6/4) | 6 (2/2/2) | 6 (2/2/2) | 6 (2/2/2) | 18 (6/6/6) |
| *C. dubliniensis* | *C. kefyr* | 6 (2/2/2) | 6 (2/2/2) | 6 (2/2/2) | 18 (6/6/6) | 6 (2/2/2) | 6 (2/2/2) | 6 (2/2/2) | 18 (6/6/6) | 4 (1/2/1) | 3 (1/1/1) | 6 (2/2/2) | 13 (4/5/4) |
| *C. dubliniensis* | *C. krusei* | 6 (2/2/2) | 6 (2/2/2) | 6 (2/2/2) | 18 (6/6/6) | 6 (2/2/2) | 6 (2/2/2) | 6 (2/2/2) | 18 (6/6/6) | 6 (2/2/2) | 6 (2/2/2) | 6 (2/2/2) | 18 (6/6/6) |
| *C. dubliniensis* | *C. lusitaniae* | 5 (1/2/2) | 6 (2/2/2) | 6 (2/2/2) | 17 (5/6/6) | 3 (1/1/1) | 3 (1/1/1) | 6 (2/2/2) | 12 (4/4/4) | 5 (1/2/2) | 5 (2/2/1) | 5 (2/2/1) | 15 (5/6/4) |
| *C. dubliniensis* | *C. nivariensis* | 4 (1/2/1) | 5 (1/2/2) | 5 (1/2/2) | 14 (3/6/5) | 6 (2/2/2) | 6 (2/2/2) | 6 (2/2/2) | 18 (6/6/6) | 4 (1/2/1) | 5 (2/2/1) | 5 (2/2/1) | 14 (5/6/3) |
| *C. dubliniensis* | *C. norvegensis* | 6 (2/2/2) | 6 (2/2/2) | 6 (2/2/2) | 18 (6/6/6) | 6 (2/2/2) | 6 (2/2/2) | 6 (2/2/2) | 18 (6/6/6) | 6 (2/2/2) | 6 (2/2/2) | 6 (2/2/2) | 18 (6/6/6) |
| *C. dubliniensis* | *C. parapsilosis* | 6 (2/2/2) | 6 (2/2/2) | 6 (2/2/2) | 18 (6/6/6) | 6 (2/2/2) | 6 (2/2/2) | 6 (2/2/2) | 18 (6/6/6) | 6 (2/2/2) | 5 (2/1/2) | 6 (2/2/2) | 17 (6/5/6) |
| *C. dubliniensis* | *C. pararugosa* | 3 (1/1/1) | 5 (2/2/1) | 5 (2/2/1) | 13 (5/5/3) | 3 (1/2/–) | 6 (2/2/2) | 6 (2/2/2) | 15 (5/6/4) | 3 (1/1/1) | 6 (2/2/2) | 6 (2/2/2) | 15 (5/5/5) |
| *C. dubliniensis* | *C. pelliculosa* | 3 (1/1/1) | 4 (1/2/1) | 4 (1/2/1) | 11 (3/5/3) | 3 (1/1/1) | 6 (2/2/2) | 6 (2/2/2) | 15 (5/5/5) | 3 (1/1/1) | 5 (2/2/1) | 5 (2/2/1) | 13 (5/5/3) |
| *C. dubliniensis* | *C. robusta* | 6 (2/2/2) | 6 (2/2/2) | 6 (2/2/2) | 18 (6/6/6) | 6 (2/2/2) | 6 (2/2/2) | 6 (2/2/2) | 18 (6/6/6) | 3 (1/1/1) | 5 (1/2/2) | 5 (1/2/2) | 13 (3/5/5) |
| *C. dubliniensis* | *C. sorbosa* | 3 (1/1/1) | 6 (2/2/2) | 6 (2/2/2) | 15 (5/5/5) | 3 (1/1/1) | 3 (1/1/1) | 4 (1/2/1) | 10 (3/4/3) | 4 (1/2/1) | 6 (2/2/2) | 6 (2/2/2) | 16 (5/6/5) |
| *C. dubliniensis* | *C. tropicalis* | 6 (2/2/2) | 4 (1/2/1) | 4 (1/2/1) | 14 (4/6/4) | 6 (2/2/2) | 6 (2/2/2) | 6 (2/2/2) | 18 (6/6/6) | 4 (1/2/1) | 3 (1/1/1) | 4 (1/2/1) | 11 (3/5/3) |
| *C. dubliniensis* | *C. utilis* | 6 (2/2/2) | 7 (2/3/2) | 6 (2/2/2) | 19 (6/7/6) | 6 (2/2/2) | 6 (2/2/2) | 6 (2/2/2) | 18 (6/6/6) | 6 (2/2/2) | 6 (2/2/2) | 6 (2/2/2) | 18 (6/6/6) |
| *C. glabrata* | – | 6 (2/2/2) | 6 (2/2/2) | 6 (2/2/2) | 18 (6/6/6) | 4 (1/2/1) | 5 (1/2/2) | 5 (1/2/2) | 14 (3/6/5) | 5 (1/2/2) | 6 (2/2/2) | 6 (2/2/2) | 17 (5/6/6) |
| *C. glabrata* | *C. guilliermondii* | 6 (2/2/2) | 6 (2/2/2) | 6 (2/2/2) | 18 (6/6/6) | 6 (2/2/2) | 7 (2/3/2) | 6 (2/2/2) | 19 (6/7/6) | 5 (1/2/2) | 6 (2/2/2) | 6 (2/2/2) | 17 (5/6/6) |
| *C. glabrata* | *C. incospicua* | 6 (2/2/2) | 7 (2/3/2) | 7 (2/3/2) | 20 (6/8/6) | 3 (1/1/1) | 5 (1/2/2) | 5 (1/2/2) | 13 (3/5/5) | 6 (2/2/2) | 6 (2/2/2) | 6 (2/2/2) | 18 (6/6/6) |
| *C. glabrata* | *C. kefyr* | 6 (2/2/2) | 7 (2/3/2) | 6 (2/2/2) | 19 (6/7/6) | 5 (1/2/2) | 5 (1/2/2) | 5 (1/2/2) | 15 (3/6/6) | 4 (1/2/1) | 4 (1/2/1) | 4 (1/2/1) | 12 (3/6/3) |
| *C. glabrata* | *C. krusei* | 8 (2/3/3) | 7 (2/2/3) | 7 (2/2/3) | 22 (6/7/9) | 6 (2/2/2) | 6 (2/2/2) | 6 (2/2/2) | 18 (6/6/6) | 6 (2/2/2) | 6 (2/2/2) | 6 (2/2/2) | 18 (6/6/6) |
| *C. glabrata* | *C. lusitaniae* | 6 (2/2/2) | 5 (1/2/2) | 5 (1/2/2) | 16 (4/6/6) | 4 (1/2/1) | 5 (1/2/2) | 5 (1/2/2) | 14 (3/6/5) | 5 (1/2/2) | 4 (1/2/1) | 4 (1/2/1) | 13 (3/6/4) |
| *C. glabrata* | *C. nivariensis* | 6 (2/2/2) | 7 (2/3/2) | 6 (2/2/2) | 19 (6/7/6) | 4 (1/2/1) | 5 (1/2/2) | 5 (1/2/2) | 14 (3/6/5) | 7 (2/3/2) | 6 (2/2/2) | 6 (2/2/2) | 19 (6/7/6) |
| *C. glabrata* | *C. norvegensis* | 6 (2/2/2) | 7 (2/3/2) | 7 (2/3/2) | 20 (6/8/6) | 5 (1/2/2) | 5 (1/2/2) | 5 (1/2/2) | 15 (3/6/6) | 6 (2/2/2) | 6 (2/2/2) | 6 (2/2/2) | 18 (6/6/6) |
| *C. glabrata* | *C. parapsilosis* | 6 (2/2/2) | 7 (2/2/3) | 7 (2/2/3) | 20 (6/6/8) | 6 (2/2/2) | 6 (2/2/2) | 6 (2/2/2) | 18 (6/6/6) | 6 (2/2/2) | 6 (2/2/2) | 6 (2/2/2) | 18 (6/6/6) |
| *C. glabrata* | *C. pararugosa* | 6 (2/2/2) | 7 (2/3/2) | 5 (2/1/2) | 18 (6/6/6) | 3 (1/1/1) | 5 (1/2/2) | 5 (1/2/2) | 13 (3/5/5) | 6 (2/2/2) | 7 (2/3/2) | 6 (2/2/2) | 19 (6/7/6) |
| *C. glabrata* | *C. pelliculosa* | 6 (2/2/2) | 6 (2/2/2) | 6 (2/2/2) | 18 (6/6/6) | 2 (1/1/–) | 5 (1/2/2) | 5 (1/2/2) | 12 (3/5/4) | 6 (2/2/2) | 6 (2/2/2) | 6 (2/2/2) | 18 (6/6/6) |
| *C. glabrata* | *C. robusta* | 7 (2/3/2) | 8 (2/3/3) | 8 (2/3/3) | 23 (6/9/8) | 6 (2/2/2) | 6 (2/2/2) | 6 (2/2/2) | 18 (6/6/6) | 5 (1/2/2) | 4 (1/2/1) | 4 (1/2/1) | 13 (3/6/4) |
| *C. glabrata* | *C. sorbosa* | 6 (2/2/2) | 7 (2/3/2) | 6 (2/2/2) | 19 (6/7/6) | 2 (1/1/–) | 5 (1/2/2) | 5 (1/2/2) | 12 (3/5/4) | 4 (1/2/1) | 6 (2/2/2) | 6 (2/2/2) | 16 (5/6/5) |
| *C. glabrata* | *C. tropicalis* | 6 (2/2/2) | 7 (2/3/2) | 6 (2/2/2) | 19 (6/7/6) | 6 (2/2/2) | 8 (2/3/3) | 7 (2/2/3) | 21 (6/7/8) | 6 (2/2/2) | 6 (2/2/2) | 6 (2/2/2) | 18 (6/6/6) |
| *C. glabrata* | *C. utilis* | 7 (2/3/2) | 7 (2/3/2) | 7 (2/3/2) | 21 (6/9/6) | 5 (2/2/1) | 5 (1/2/2) | 5 (1/2/2) | 15 (4/6/5) | 6 (2/2/2) | 6 (2/2/2) | 6 (2/2/2) | 18 (6/6/6) |
| *C. guilliermondii* | – | 3 (1/1/1) | 3 (1/1/1) | 3 (1/1/1) | 9 (3/3/3) | 4 (1/1/2) | 3 (1/1/1) | 3 (1/1/1) | 10 (3/3/4) | 3 (1/1/1) | 3 (1/1/1) | 3 (1/1/1) | 9 (3/3/3) |
| *C. guilliermondii* | *C. incospicua* | 6 (2/2/2) | 6 (2/2/2) | 6 (2/2/2) | 18 (6/6/6) | 4 (1/2/1) | 6 (2/2/2) | 6 (2/2/2) | 16 (5/6/5) | 5 (1/2/2) | 6 (2/2/2) | 6 (2/2/2) | 17 (5/6/6) |
| *C. guilliermondii* | *C. kefyr* | 6 (2/2/2) | 6 (2/2/2) | 6 (2/2/2) | 18 (6/6/6) | 6 (2/2/2) | 6 (2/2/2) | 6 (2/2/2) | 18 (6/6/6) | 5 (1/2/2) | 5 (2/2/1) | 5 (2/2/1) | 15 (5/6/4) |
| *C. guilliermondii* | *C. krusei* | 6 (2/2/2) | 6 (2/2/2) | 6 (2/2/2) | 18 (6/6/6) | 7 (2/3/2) | 7 (2/3/2) | 6 (2/2/2) | 20 (6/8/6) | 6 (2/2/2) | 6 (2/2/2) | 6 (2/2/2) | 18 (6/6/6) |
| *C. guilliermondii* | *C. lusitaniae* | 6 (2/2/2) | 6 (2/2/2) | 6 (2/2/2) | 18 (6/6/6) | 3 (1/1/1) | 3 (1/1/1) | 6 (2/2/2) | 12 (4/4/4) | 5 (1/2/2) | 4 (1/2/1) | 4 (1/2/1) | 13 (3/6/4) |
| *C. guilliermondii* | *C. nivariensis* | 6 (2/2/2) | 5 (1/2/2) | 5 (1/2/2) | 16 (4/6/6) | 6 (2/2/2) | 6 (2/2/2) | 6 (2/2/2) | 18 (6/6/6) | 6 (2/2/2) | 5 (1/2/2) | 5 (1/2/2) | 16 (4/6/6) |
| *C. guilliermondii* | *C. norvegensis* | 6 (2/2/2) | 6 (2/2/2) | 6 (2/2/2) | 18 (6/6/6) | 6 (2/2/2) | 6 (2/2/2) | 6 (2/2/2) | 18 (6/6/6) | 6 (2/2/2) | 6 (2/2/2) | 6 (2/2/2) | 18 (6/6/6) |
| *C. guilliermondii* | *C. parapsilosis* | 6 (2/2/2) | 6 (2/2/2) | 6 (2/2/2) | 18 (6/6/6) | 4 (1/2/1) | 5 (1/2/2) | 5 (1/2/2) | 14 (3/6/5) | 6 (2/2/2) | 6 (2/2/2) | 6 (2/2/2) | 18 (6/6/6) |
| *C. guilliermondii* | *C. pararugosa* | 3 (1/1/1) | 6 (2/2/2) | 6 (2/2/2) | 15 (5/5/5) | 4 (1/1/2) | 3 (1/1/1) | 3 (1/1/1) | 10 (3/3/4) | 3 (1/1/1) | 5 (2/1/2) | 6 (2/2/2) | 14 (5/4/5) |
| *C. guilliermondii* | *C. pelliculosa* | 3 (1/1/1) | 3 (1/1/1) | 6 (2/2/2) | 12 (4/4/4) | 3 (1/1/1) | 6 (2/2/2) | 6 (2/2/2) | 15 (5/5/5) | 3 (1/1/1) | 5 (1/2/2) | 5 (1/2/2) | 13 (3/5/5) |
| *C. guilliermondii* | *C. robusta* | 6 (2/2/2) | 6 (2/2/2) | 6 (2/2/2) | 18 (6/6/6) | 4 (1/2/1) | 6 (2/2/2) | 6 (2/2/2) | 16 (5/6/5) | 6 (2/2/2) | 6 (2/2/2) | 6 (2/2/2) | 18 (6/6/6) |
| *C. guilliermondii* | *C. sorbosa* | 3 (1/1/1) | 6 (2/2/2) | 6 (2/2/2) | 15 (5/5/5) | 4 (1/2/1) | 3 (1/1/1) | 3 (1/1/1) | 10 (3/4/3) | 3 (1/1/1) | 6 (2/2/2) | 6 (2/2/2) | 15 (5/5/5) |
| *C. guilliermondii* | *C. tropicalis* | 6 (2/2/2) | 6 (2/2/2) | 6 (2/2/2) | 18 (6/6/6) | 6 (2/2/2) | 6 (2/2/2) | 6 (2/2/2) | 18 (6/6/6) | 6 (2/2/2) | 6 (2/2/2) | 6 (2/2/2) | 18 (6/6/6) |
| *C. guilliermondii* | *C. utilis* | 6 (2/2/2) | 6 (2/2/2) | 6 (2/2/2) | 18 (6/6/6) | 5 (2/2/1) | 6 (2/2/2) | 6 (2/2/2) | 17 (6/6/5) | 5 (2/2/1) | 6 (2/2/2) | 6 (2/2/2) | 17 (6/6/5) |
| *C. incospicua* | – | 3 (1/1/1) | 5 (1/2/2) | 5 (1/2/2) | 13 (3/5/5) | 0 (0/0/0) | 0 (0/0/0) | 2 (–/1/1) | 2 (0/1/1) | 3 (1/1/1) | 3 (1/1/1) | 3 (1/1/1) | 9 (3/3/3) |
| *C. incospicua* | *C. kefyr* | 6 (2/2/2) | 6 (2/2/2) | 6 (2/2/2) | 18 (6/6/6) | 6 (2/2/2) | 7 (2/2/3) | 7 (2/2/3) | 20 (6/6/8) | 6 (2/2/2) | 6 (2/2/2) | 6 (2/2/2) | 18 (6/6/6) |
| *C. incospicua* | *C. krusei* | 8 (2/3/3) | 8 (2/3/3) | 7 (2/2/3) | 23 (6/8/9) | 6 (2/2/2) | 6 (2/2/2) | 6 (2/2/2) | 18 (6/6/6) | 7 (2/3/2) | 7 (2/3/2) | 6 (2/2/2) | 20 (6/8/6) |
| *C. incospicua* | *C. lusitaniae* | 6 (2/2/2) | 6 (2/2/2) | 6 (2/2/2) | 18 (6/6/6) | 2 (1/1/–) | 3 (1/1/1) | 4 (1/2/1) | 9 (3/4/2) | 6 (2/2/2) | 6 (2/2/2) | 6 (2/2/2) | 18 (6/6/6) |
| *C. incospicua* | *C. nivariensis* | 6 (2/2/2) | 6 (2/2/2) | 6 (2/2/2) | 18 (6/6/6) | 3 (1/1/1) | 4 (1/2/1) | 4 (1/2/1) | 11 (3/5/3) | 6 (2/2/2) | 6 (2/2/2) | 6 (2/2/2) | 18 (6/6/6) |
| *C. incospicua* | *C. norvegensis* | 6 (2/2/2) | 6 (2/2/2) | 6 (2/2/2) | 18 (6/6/6) | 5 (1/2/2) | 5 (1/2/2) | 5 (1/2/2) | 15 (3/6/6) | 6 (2/2/2) | 6 (2/2/2) | 6 (2/2/2) | 18 (6/6/6) |
| *C. incospicua* | *C. parapsilosis* | 6 (2/2/2) | 6 (2/2/2) | 6 (2/2/2) | 18 (6/6/6) | 6 (2/2/2) | 6 (2/2/2) | 6 (2/2/2) | 18 (6/6/6) | 6 (2/2/2) | 6 (2/2/2) | 6 (2/2/2) | 18 (6/6/6) |
| *C. incospicua* | *C. pararugosa* | 4 (1/1/2) | 6 (2/2/2) | 6 (2/2/2) | 16 (5/5/6) | 0 (0/0/0) | 6 (2/2/2) | 6 (2/2/2) | 12 (4/4/4) | 3 (1/1/1) | 6 (2/2/2) | 6 (2/2/2) | 15 (5/5/5) |
| *C. incospicua* | *C. pelliculosa* | 4 (1/1/2) | 6 (2/2/2) | 6 (2/2/2) | 16 (5/5/6) | 0 (0/0/0) | 0 (0/0/0) | 2 (–/1/1) | 2 (0/1/1) | 3 (1/1/1) | 6 (2/2/2) | 6 (2/2/2) | 15 (5/5/5) |
| *C. incospicua* | *C. robusta* | 6 (2/2/2) | 6 (2/2/2) | 6 (2/2/2) | 18 (6/6/6) | 6 (2/2/2) | 6 (2/2/2) | 6 (2/2/2) | 18 (6/6/6) | 6 (2/2/2) | 6 (2/2/2) | 6 (2/2/2) | 18 (6/6/6) |
| *C. incospicua* | *C. sorbosa* | 4 (1/2/1) | 6 (2/2/2) | 6 (2/2/2) | 16 (5/6/5) | 0 (0/0/0) | 0 (0/0/0) | 3 (1/1/1) | 3 (1/1/1) | 4 (1/2/1) | 6 (2/2/2) | 6 (2/2/2) | 16 (5/6/5) |
| *C. incospicua* | *C. tropicalis* | 6 (2/2/2) | 6 (2/2/2) | 6 (2/2/2) | 18 (6/6/6) | 3 (1/1/1) | 4 (1/2/1) | 4 (1/2/1) | 11 (3/5/3) | 6 (2/2/2) | 6 (2/2/2) | 6 (2/2/2) | 18 (6/6/6) |
| *C. incospicua* | *C. utilis* | 5 (1/2/2) | 6 (2/2/2) | 6 (2/2/2) | 17 (5/6/6) | 4 (2/2/–) | 6 (2/2/2) | 6 (2/2/2) | 16 (6/6/4) | 5 (1/2/2) | 5 (1/2/2) | 5 (1/2/2) | 15 (3/6/6) |
| *C. kefyr* | – | 3 (1/1/1) | 4 (1/2/1) | 4 (1/2/1) | 11 (3/5/3) | 3 (1/1/1) | 3 (1/1/1) | 4 (1/2/1) | 10 (3/4/3) | 4 (1/2/1) | 6 (1/3/2) | 5 (1/2/2) | 15 (3/7/5) |
| *C. kefyr* | *C. krusei* | 6 (2/2/2) | 6 (2/2/2) | 6 (2/2/2) | 18 (6/6/6) | 6 (2/2/2) | 6 (2/2/2) | 6 (2/2/2) | 18 (6/6/6) | 6 (2/2/2) | 6 (2/2/2) | 6 (2/2/2) | 18 (6/6/6) |
| *C. kefyr* | *C. lusitaniae* | 4 (1/2/1) | 5 (1/2/2) | 5 (1/2/2) | 14 (3/6/5) | 6 (2/2/2) | 5 (1/2/2) | 5 (1/2/2) | 16 (4/6/6) | 3 (1/1/1) | 4 (1/2/1) | 4 (1/2/1) | 11 (3/5/3) |
| *C. kefyr* | *C. nivariensis* | 5 (1/2/2) | 6 (2/2/2) | 6 (2/2/2) | 17 (5/6/6) | 3 (1/1/1) | 5 (1/2/2) | 5 (1/2/2) | 13 (3/5/5) | 4 (1/2/1) | 5 (1/2/2) | 5 (1/2/2) | 14 (3/6/5) |
| *C. kefyr* | *C. norvegensis* | 6 (2/2/2) | 6 (2/2/2) | 6 (2/2/2) | 18 (6/6/6) | 4 (1/1/2) | 5 (1/2/2) | 5 (1/2/2) | 14 (3/5/6) | 6 (2/2/2) | 5 (2/2/1) | 5 (2/2/1) | 16 (6/6/4) |
| *C. kefyr* | *C. parapsilosis* | 5 (2/2/1) | 6 (2/2/2) | 6 (2/2/2) | 17 (6/6/5) | 6 (2/2/2) | 6 (2/2/2) | 6 (2/2/2) | 18 (6/6/6) | 6 (2/2/2) | 6 (2/2/2) | 6 (2/2/2) | 18 (6/6/6) |
| *C. kefyr* | *C. pararugosa* | 6 (2/2/2) | 6 (2/2/2) | 6 (2/2/2) | 18 (6/6/6) | 3 (1/1/1) | 5 (1/2/2) | 5 (1/2/2) | 13 (3/5/5) | 6 (2/2/2) | 6 (2/2/2) | 6 (2/2/2) | 18 (6/6/6) |
| *C. kefyr* | *C. pelliculosa* | 6 (2/2/2) | 6 (2/2/2) | 6 (2/2/2) | 18 (6/6/6) | 6 (2/2/2) | 6 (2/2/2) | 6 (2/2/2) | 18 (6/6/6) | 5 (1/2/2) | 6 (2/2/2) | 6 (2/2/2) | 17 (5/6/6) |
| *C. kefyr* | *C. robusta* | 5 (1/2/2) | 5 (1/2/2) | 5 (1/2/2) | 15 (3/6/6) | 6 (2/2/2) | 6 (2/2/2) | 6 (2/2/2) | 18 (6/6/6) | 5 (1/2/2) | 5 (1/2/2) | 5 (1/2/2) | 15 (3/6/6) |
| *C. kefyr* | *C. sorbosa* | 6 (2/2/2) | 6 (2/2/2) | 6 (2/2/2) | 18 (6/6/6) | 5 (1/2/2) | 5 (1/2/2) | 5 (1/2/2) | 15 (3/6/6) | 6 (2/2/2) | 6 (2/2/2) | 6 (2/2/2) | 18 (6/6/6) |
| *C. kefyr* | *C. tropicalis* | 4 (1/1/2) | 4 (1/2/1) | 4 (1/2/1) | 12 (3/5/4) | 6 (2/2/2) | 6 (2/2/2) | 6 (2/2/2) | 18 (6/6/6) | 3 (1/1/1) | 3 (1/1/1) | 6 (2/2/2) | 12 (4/4/4) |
| *C. kefyr* | *C. utilis* | 6 (2/2/2) | 6 (2/2/2) | 6 (2/2/2) | 18 (6/6/6) | 5 (1/2/2) | 5 (1/2/2) | 5 (1/2/2) | 15 (3/6/6) | 6 (2/2/2) | 6 (2/2/2) | 6 (2/2/2) | 18 (6/6/6) |
| *C. krusei* | – | 3 (1/1/1) | 3 (1/1/1) | 3 (1/1/1) | 9 (3/3/3) | 3 (1/1/1) | 3 (1/1/1) | 3 (1/1/1) | 9 (3/3/3) | 3 (1/1/1) | 3 (1/1/1) | 3 (1/1/1) | 9 (3/3/3) |
| *C. krusei* | *C. lusitaniae* | 6 (2/2/2) | 6 (2/2/2) | 6 (2/2/2) | 18 (6/6/6) | 6 (2/2/2) | 7 (2/3/2) | 7 (2/3/2) | 20 (6/8/6) | 6 (2/2/2) | 6 (2/2/2) | 6 (2/2/2) | 18 (6/6/6) |
| *C. krusei* | *C. nivariensis* | 6 (2/2/2) | 6 (2/2/2) | 6 (2/2/2) | 18 (6/6/6) | 6 (2/2/2) | 6 (2/2/2) | 6 (2/2/2) | 18 (6/6/6) | 6 (2/2/2) | 6 (2/2/2) | 6 (2/2/2) | 18 (6/6/6) |
| *C. krusei* | *C. norvegensis* | 6 (2/2/2) | 6 (2/2/2) | 6 (2/2/2) | 18 (6/6/6) | 7 (2/2/3) | 6 (2/2/2) | 6 (2/2/2) | 19 (6/6/7) | 6 (2/2/2) | 7 (2/2/3) | 7 (2/2/3) | 20 (6/6/8) |
| *C. krusei* | *C. parapsilosis* | 6 (2/2/2) | 6 (2/2/2) | 6 (2/2/2) | 18 (6/6/6) | 5 (1/2/2) | 6 (2/2/2) | 6 (2/2/2) | 17 (5/6/6) | 5 (2/2/1) | 6 (2/2/2) | 6 (2/2/2) | 17 (6/6/5) |
| *C. krusei* | *C. pararugosa* | 4 (1/2/1) | 6 (2/2/2) | 6 (2/2/2) | 16 (5/6/5) | 3 (1/1/1) | 4 (1/2/1) | 4 (1/2/1) | 11 (3/5/3) | 5 (2/2/1) | 6 (2/2/2) | 6 (2/2/2) | 17 (6/6/5) |
| *C. krusei* | *C. pelliculosa* | 5 (1/2/2) | 5 (1/2/2) | 5 (1/2/2) | 15 (3/6/6) | 4 (1/2/1) | 5 (1/2/2) | 5 (1/2/2) | 14 (3/6/5) | 5 (1/2/2) | 5 (1/2/2) | 5 (1/2/2) | 15 (3/6/6) |
| *C. krusei* | *C. robusta* | 6 (2/2/2) | 6 (2/2/2) | 6 (2/2/2) | 18 (6/6/6) | 6 (2/2/2) | 7 (2/2/3) | 7 (2/2/3) | 20 (6/6/8) | 6 (2/2/2) | 6 (2/2/2) | 6 (2/2/2) | 18 (6/6/6) |
| *C. krusei* | *C. sorbosa* | 7 (2/3/2) | 6 (2/2/2) | 5 (2/1/2) | 18 (6/6/6) | 6 (2/2/2) | 6 (2/2/2) | 6 (2/2/2) | 18 (6/6/6) | 5 (1/2/2) | 4 (1/2/1) | 4 (1/2/1) | 13 (3/6/4) |
| *C. krusei* | *C. tropicalis* | 6 (2/2/2) | 5 (2/2/1) | 5 (2/2/1) | 16 (6/6/4) | 9 (3/2/4) | 6 (2/2/2) | 6 (2/2/2) | 21 (7/6/8) | 6 (2/2/2) | 6 (2/2/2) | 6 (2/2/2) | 18 (6/6/6) |
| *C. krusei* | *C. utilis* | 6 (2/2/2) | 6 (2/2/2) | 6 (2/2/2) | 18 (6/6/6) | 6 (2/2/2) | 6 (2/2/2) | 6 (2/2/2) | 18 (6/6/6) | 6 (2/2/2) | 6 (2/2/2) | 6 (2/2/2) | 18 (6/6/6) |
| *C. lusitaniae* | – | 2 (–/1/1) | 3 (1/1/1) | 3 (1/1/1) | 8 (2/3/3) | 0 (0/0/0) | 1 (–/1/–) | 3 (1/1/1) | 4 (1/2/1) | 3 (1/1/1) | 3 (1/1/1) | 3 (1/1/1) | 9 (3/3/3) |
| *C. lusitaniae* | *C. nivariensis* | 6 (2/2/2) | 6 (2/2/2) | 6 (2/2/2) | 18 (6/6/6) | 3 (1/1/1) | 3 (1/1/1) | 6 (2/2/2) | 12 (4/4/4) | 6 (2/2/2) | 5 (1/2/2) | 5 (1/2/2) | 16 (4/6/6) |
| *C. lusitaniae* | *C. norvegensis* | 6 (2/2/2) | 6 (2/2/2) | 6 (2/2/2) | 18 (6/6/6) | 7 (2/2/3) | 6 (2/2/2) | 6 (2/2/2) | 19 (6/6/7) | 6 (2/2/2) | 6 (2/2/2) | 6 (2/2/2) | 18 (6/6/6) |
| *C. lusitaniae* | *C. parapsilosis* | 6 (2/2/2) | 6 (2/2/2) | 6 (2/2/2) | 18 (6/6/6) | 5 (1/2/2) | 5 (1/2/2) | 5 (1/2/2) | 15 (3/6/6) | 6 (2/2/2) | 6 (2/2/2) | 6 (2/2/2) | 18 (6/6/6) |
| *C. lusitaniae* | *C. pararugosa* | 3 (1/1/1) | 6 (2/2/2) | 6 (2/2/2) | 15 (5/5/5) | 0 (0/0/0) | 3 (1/1/1) | 6 (2/2/2) | 9 (3/3/3) | 4 (1/2/1) | 6 (2/2/2) | 6 (2/2/2) | 16 (5/6/5) |
| *C. lusitaniae* | *C. pelliculosa* | 3 (1/1/1) | 3 (1/1/1) | 6 (2/2/2) | 12 (4/4/4) | 0 (0/0/0) | 6 (2/2/2) | 6 (2/2/2) | 12 (4/4/4) | 3 (1/1/1) | 4 (1/2/1) | 4 (1/2/1) | 11 (3/5/3) |
| *C. lusitaniae* | *C. robusta* | 5 (1/2/2) | 5 (1/2/2) | 5 (1/2/2) | 15 (3/6/6) | 6 (2/2/2) | 6 (2/2/2) | 6 (2/2/2) | 18 (6/6/6) | 4 (1/2/1) | 4 (1/2/1) | 4 (1/2/1) | 12 (3/6/3) |
| *C. lusitaniae* | *C. sorbosa* | 4 (1/1/2) | 6 (2/2/2) | 6 (2/2/2) | 16 (5/5/6) | 0 (0/0/0) | 3 (1/1/1) | 6 (2/2/2) | 9 (3/3/3) | 5 (2/2/1) | 6 (2/2/2) | 6 (2/2/2) | 17 (6/6/5) |
| *C. lusitaniae* | *C. tropicalis* | 6 (2/2/2) | 6 (2/2/2) | 6 (2/2/2) | 18 (6/6/6) | 3 (1/1/1) | 3 (1/1/1) | 6 (2/2/2) | 12 (4/4/4) | 5 (1/2/2) | 6 (2/2/2) | 6 (2/2/2) | 17 (5/6/6) |
| *C. lusitaniae* | *C. utilis* | 6 (2/2/2) | 7 (2/3/2) | 6 (2/2/2) | 19 (6/7/6) | 6 (2/2/2) | 6 (2/2/2) | 6 (2/2/2) | 18 (6/6/6) | 5 (1/2/2) | 6 (2/2/2) | 6 (2/2/2) | 17 (5/6/6) |
| *C. nivariensis* | – | 3 (1/1/1) | 3 (1/1/1) | 3 (1/1/1) | 9 (3/3/3) | 4 (1/2/1) | 5 (1/2/2) | 5 (1/2/2) | 14 (3/6/5) | 3 (1/1/1) | 3 (1/1/1) | 3 (1/1/1) | 9 (3/3/3) |
| *C. nivariensis* | *C. norvegensis* | 6 (2/2/2) | 5 (2/1/2) | 6 (2/2/2) | 17 (6/5/6) | 4 (1/1/2) | 4 (1/1/2) | 4 (1/1/2) | 12 (3/3/6) | 6 (2/2/2) | 6 (2/2/2) | 6 (2/2/2) | 18 (6/6/6) |
| *C. nivariensis* | *C. parapsilosis* | 6 (2/2/2) | 6 (2/2/2) | 6 (2/2/2) | 18 (6/6/6) | 5 (2/2/1) | 6 (2/2/2) | 6 (2/2/2) | 17 (6/6/5) | 3 (1/1/1) | 6 (2/2/2) | 6 (2/2/2) | 15 (5/5/5) |
| *C. nivariensis* | *C. pararugosa* | 4 (1/1/2) | 5 (1/2/2) | 5 (1/2/2) | 14 (3/5/6) | 3 (1/1/1) | 5 (1/2/2) | 5 (1/2/2) | 13 (3/5/5) | 3 (1/1/1) | 4 (1/2/1) | 4 (1/2/1) | 11 (3/5/3) |
| *C. nivariensis* | *C. pelliculosa* | 3 (1/1/1) | 3 (1/1/1) | 4 (1/2/1) | 10 (3/4/3) | 3 (1/1/1) | 3 (1/1/1) | 3 (1/1/1) | 9 (3/3/3) | 3 (1/1/1) | 3 (1/1/1) | 4 (1/2/1) | 10 (3/4/3) |
| *C. nivariensis* | *C. robusta* | 6 (2/2/2) | 6 (2/2/2) | 6 (2/2/2) | 18 (6/6/6) | 6 (2/2/2) | 6 (2/2/2) | 6 (2/2/2) | 18 (6/6/6) | 5 (1/2/2) | 5 (2/2/1) | 5 (2/2/1) | 15 (5/6/4) |
| *C. nivariensis* | *C. sorbosa* | 4 (1/2/1) | 5 (1/2/2) | 5 (1/2/2) | 14 (3/6/5) | 3 (1/1/1) | 3 (1/1/1) | 4 (1/2/1) | 10 (3/4/3) | 4 (1/2/1) | 6 (2/2/2) | 6 (2/2/2) | 16 (5/6/5) |
| *C. nivariensis* | *C. tropicalis* | 6 (2/2/2) | 6 (2/2/2) | 6 (2/2/2) | 18 (6/6/6) | 6 (2/2/2) | 6 (2/2/2) | 6 (2/2/2) | 18 (6/6/6) | 3 (1/1/1) | 3 (1/1/1) | 6 (2/2/2) | 12 (4/4/4) |
| *C. nivariensis* | *C. utilis* | 7 (2/2/3) | 7 (2/2/3) | 7 (2/2/3) | 21 (6/6/9) | 4 (1/2/1) | 6 (2/2/2) | 6 (2/2/2) | 16 (5/6/5) | 6 (2/2/2) | 6 (2/2/2) | 6 (2/2/2) | 18 (6/6/6) |
| *C. norvegensis* | – | 3 (1/1/1) | 3 (1/1/1) | 3 (1/1/1) | 9 (3/3/3) | 4 (1/1/2) | 3 (1/1/1) | 3 (1/1/1) | 10 (3/3/4) | 3 (1/1/1) | 4 (1/1/2) | 5 (1/2/2) | 12 (3/4/5) |
| *C. norvegensis* | *C. parapsilosis* | 5 (2/2/1) | 6 (2/2/2) | 6 (2/2/2) | 17 (6/6/5) | 6 (2/2/2) | 6 (2/2/2) | 6 (2/2/2) | 18 (6/6/6) | 5 (2/2/1) | 6 (2/2/2) | 6 (2/2/2) | 17 (6/6/5) |
| *C. norvegensis* | *C. pararugosa* | 6 (2/2/2) | 6 (2/2/2) | 6 (2/2/2) | 18 (6/6/6) | 4 (1/1/2) | 5 (1/2/2) | 5 (1/2/2) | 14 (3/5/6) | 5 (2/2/1) | 6 (2/2/2) | 6 (2/2/2) | 17 (6/6/5) |
| *C. norvegensis* | *C. pelliculosa* | 6 (2/2/2) | 6 (2/2/2) | 6 (2/2/2) | 18 (6/6/6) | 5 (1/2/2) | 6 (2/2/2) | 6 (2/2/2) | 17 (5/6/6) | 6 (2/2/2) | 6 (2/2/2) | 6 (2/2/2) | 18 (6/6/6) |
| *C. norvegensis* | *C. robusta* | 6 (2/2/2) | 6 (2/2/2) | 6 (2/2/2) | 18 (6/6/6) | 7 (2/2/3) | 6 (2/2/2) | 6 (2/2/2) | 19 (6/6/7) | 6 (2/2/2) | 6 (2/2/2) | 6 (2/2/2) | 18 (6/6/6) |
| *C. norvegensis* | *C. sorbosa* | 6 (2/2/2) | 6 (2/2/2) | 6 (2/2/2) | 18 (6/6/6) | 5 (1/2/2) | 6 (2/2/2) | 6 (2/2/2) | 17 (5/6/6) | 5 (1/2/2) | 6 (2/2/2) | 6 (2/2/2) | 17 (5/6/6) |
| *C. norvegensis* | *C. tropicalis* | 6 (2/2/2) | 6 (2/2/2) | 6 (2/2/2) | 18 (6/6/6) | 7 (2/2/3) | 6 (2/2/2) | 6 (2/2/2) | 19 (6/6/7) | 6 (2/2/2) | 6 (2/2/2) | 6 (2/2/2) | 18 (6/6/6) |
| *C. norvegensis* | *C. utilis* | 5 (2/2/1) | 6 (2/2/2) | 6 (2/2/2) | 17 (6/6/5) | 5 (1/2/2) | 4 (1/1/2) | 5 (1/2/2) | 14 (3/5/6) | 6 (2/2/2) | 6 (2/2/2) | 6 (2/2/2) | 18 (6/6/6) |
| *C. parapsilosis* | – | 3 (1/1/1) | 3 (1/1/1) | 3 (1/1/1) | 9 (3/3/3) | 3 (1/1/1) | 3 (1/1/1) | 3 (1/1/1) | 9 (3/3/3) | 3 (1/1/1) | 3 (1/1/1) | 3 (1/1/1) | 9 (3/3/3) |
| *C. parapsilosis* | *C. pararugosa* | 6 (2/2/2) | 5 (1/2/2) | 5 (1/2/2) | 16 (4/6/6) | 3 (1/1/1) | 3 (1/1/1) | 6 (2/2/2) | 12 (4/4/4) | 6 (2/2/2) | 6 (2/2/2) | 6 (2/2/2) | 18 (6/6/6) |
| *C. parapsilosis* | *C. pelliculosa* | 6 (2/2/2) | 6 (2/2/2) | 6 (2/2/2) | 18 (6/6/6) | 6 (2/2/2) | 6 (2/2/2) | 6 (2/2/2) | 18 (6/6/6) | 6 (2/2/2) | 6 (2/2/2) | 6 (2/2/2) | 18 (6/6/6) |
| *C. parapsilosis* | *C. robusta* | 6 (2/2/2) | 6 (2/2/2) | 6 (2/2/2) | 18 (6/6/6) | 6 (2/2/2) | 6 (2/2/2) | 6 (2/2/2) | 18 (6/6/6) | 6 (2/2/2) | 6 (2/2/2) | 6 (2/2/2) | 18 (6/6/6) |
| *C. parapsilosis* | *C. sorbosa* | 6 (2/2/2) | 7 (2/2/3) | 7 (2/2/3) | 20 (6/6/8) | 5 (1/2/2) | 6 (2/2/2) | 6 (2/2/2) | 17 (5/6/6) | 5 (1/2/2) | 6 (2/2/2) | 6 (2/2/2) | 17 (5/6/6) |
| *C. parapsilosis* | *C. tropicalis* | 6 (2/2/2) | 6 (2/2/2) | 6 (2/2/2) | 18 (6/6/6) | 6 (2/2/2) | 6 (2/2/2) | 6 (2/2/2) | 18 (6/6/6) | 6 (2/2/2) | 6 (2/2/2) | 6 (2/2/2) | 18 (6/6/6) |
| *C. parapsilosis* | *C. utilis* | 6 (2/2/2) | 6 (2/2/2) | 6 (2/2/2) | 18 (6/6/6) | 6 (2/2/2) | 6 (2/2/2) | 6 (2/2/2) | 18 (6/6/6) | 6 (2/2/2) | 6 (2/2/2) | 6 (2/2/2) | 18 (6/6/6) |
| *C. pararugosa* | – | 0 (0/0/0) | 3 (1/1/1) | 3 (1/1/1) | 6 (2/2/2) | 0 (0/0/0) | 3 (1/1/1) | 3 (1/1/1) | 6 (2/2/2) | 0 (0/0/0) | 3 (1/1/1) | 3 (1/1/1) | 6 (2/2/2) |
| *C. pararugosa* | *C. pelliculosa* | 0 (0/0/0) | 3 (1/1/1) | 6 (2/2/2) | 9 (3/3/3) | 0 (0/0/0) | 6 (2/2/2) | 6 (2/2/2) | 12 (4/4/4) | 0 (0/0/0) | 4 (1/2/1) | 4 (1/2/1) | 8 (2/4/2) |
| *C. pararugosa* | *C. robusta* | 5 (2/2/1) | 6 (2/2/2) | 6 (2/2/2) | 17 (6/6/5) | 3 (1/1/1) | 6 (2/2/2) | 6 (2/2/2) | 15 (5/5/5) | 5 (2/2/1) | 6 (2/2/2) | 6 (2/2/2) | 17 (6/6/5) |
| *C. pararugosa* | *C. sorbosa* | 1 (–/1/–) | 6 (2/2/2) | 6 (2/2/2) | 13 (4/5/4) | 0 (0/0/0) | 2 (1/1/–) | 2 (1/1/–) | 4 (2/2/0) | 1 (–/1/–) | 4 (2/1/1) | 4 (2/1/1) | 9 (4/3/2) |
| *C. pararugosa* | *C. tropicalis* | 3 (1/1/1) | 6 (2/2/2) | 6 (2/2/2) | 15 (5/5/5) | 3 (1/1/1) | 6 (2/2/2) | 6 (2/2/2) | 15 (5/5/5) | 3 (1/1/1) | 5 (2/2/1) | 5 (2/2/1) | 13 (5/5/3) |
| *C. pararugosa* | *C. utilis* | 6 (2/2/2) | 6 (2/2/2) | 6 (2/2/2) | 18 (6/6/6) | 4 (1/2/1) | 5 (1/2/2) | 5 (1/2/2) | 14 (3/6/5) | 4 (2/2/–) | 6 (2/2/2) | 6 (2/2/2) | 16 (6/6/4) |
| *C. pelliculosa* | – | 0 (0/0/0) | 3 (1/1/1) | 5 (1/2/2) | 8 (2/3/3) | 0 (0/0/0) | 3 (1/1/1) | 3 (1/1/1) | 6 (2/2/2) | 0 (0/0/0) | 3 (1/1/1) | 4 (1/2/1) | 7 (2/3/2) |
| *C. pelliculosa* | *C. robusta* | 6 (2/2/2) | 6 (2/2/2) | 6 (2/2/2) | 18 (6/6/6) | 6 (2/2/2) | 6 (2/2/2) | 6 (2/2/2) | 18 (6/6/6) | 6 (2/2/2) | 6 (2/2/2) | 6 (2/2/2) | 18 (6/6/6) |
| *C. pelliculosa* | *C. sorbosa* | 0 (0/0/0) | 4 (1/2/1) | 5 (1/3/1) | 9 (2/5/2) | 0 (0/0/0) | 6 (2/2/2) | 6 (2/2/2) | 12 (4/4/4) | 0 (0/0/0) | 6 (2/2/2) | 6 (2/2/2) | 12 (4/4/4) |
| *C. pelliculosa* | *C. tropicalis* | 3 (1/1/1) | 4 (1/2/1) | 4 (1/2/1) | 11 (3/5/3) | 3 (1/1/1) | 6 (2/2/2) | 6 (2/2/2) | 15 (5/5/5) | 1 (–/1/–) | 5 (1/2/2) | 4 (1/1/2) | 10 (2/4/4) |
| *C. pelliculosa* | *C. utilis* | 6 (2/2/2) | 6 (2/2/2) | 6 (2/2/2) | 18 (6/6/6) | 3 (1/2/–) | 6 (2/2/2) | 6 (2/2/2) | 15 (5/6/4) | 6 (2/2/2) | 6 (2/2/2) | 6 (2/2/2) | 18 (6/6/6) |
| *C. robusta* | – | 4 (1/1/2) | 5 (2/1/2) | 5 (2/1/2) | 14 (5/3/6) | 4 (1/2/1) | 4 (1/2/1) | 4 (1/2/1) | 12 (3/6/3) | 4 (1/1/2) | 5 (1/2/2) | 5 (1/2/2) | 14 (3/5/6) |
| *C. robusta* | *C. sorbosa* | 7 (2/3/2) | 7 (2/2/3) | 8 (2/3/3) | 22 (6/8/8) | 6 (2/2/2) | 6 (2/2/2) | 6 (2/2/2) | 18 (6/6/6) | 6 (2/2/2) | 6 (2/2/2) | 6 (2/2/2) | 18 (6/6/6) |
| *C. robusta* | *C. tropicalis* | 5 (1/2/2) | 4 (1/2/1) | 4 (1/2/1) | 13 (3/6/4) | 5 (1/2/2) | 5 (1/2/2) | 5 (1/2/2) | 15 (3/6/6) | 4 (1/2/1) | 4 (1/2/1) | 3 (1/1/1) | 11 (3/5/6) |
| *C. robusta* | *C. utilis* | 6 (2/2/2) | 6 (2/2/2) | 6 (2/2/2) | 18 (6/6/6) | 6 (2/2/2) | 6 (2/2/2) | 6 (2/2/2) | 18 (6/6/6) | 6 (2/2/2) | 6 (2/2/2) | 6 (2/2/2) | 18 (6/6/6) |
| *C. sorbosa* | – | 0 (0/0/0) | 4 (1/2/1) | 4 (1/2/1) | 8 (2/4/2) | 0 (0/0/0) | 0 (0/0/0) | 3 (1/1/1) | 3 (1/1/1) | 0 (0/0/0) | 4 (1/2/1) | 4 (1/2/1) | 8 (2/4/2) |
| *C. sorbosa* | *C. tropicalis* | 3 (1/1/1) | 6 (2/2/2) | 6 (2/2/2) | 15 (5/5/5) | 3 (1/1/1) | 3 (1/1/1) | 3 (1/1/1) | 9 (3/3/3) | 3 (1/1/1) | 6 (2/2/2) | 6 (2/2/2) | 15 (5/5/5) |
| *C. sorbosa* | *C. utilis* | 7 (2/3/2) | 6 (2/2/2) | 6 (2/2/2) | 19 (6/7/6) | 4 (1/2/1) | 5 (1/2/2) | 5 (1/2/2) | 14 (3/6/5) | 6 (2/2/2) | 6 (2/2/2) | 6 (2/2/2) | 18 (6/6/6) |
| *C. tropicalis* | – | 3 (1/1/1) | 3 (1/1/1) | 3 (1/1/1) | 9 (3/3/3) | 3 (1/1/1) | 3 (1/1/1) | 3 (1/1/1) | 9 (3/3/3) | 3 (1/1/1) | 3 (1/1/1) | 3 (1/1/1) | 9 (3/3/3) |
| *C. tropicalis* | *C. utilis* | 6 (2/2/2) | 6 (2/2/2) | 6 (2/2/2) | 18 (6/6/6) | 6 (2/2/2) | 6 (2/2/2) | 6 (2/2/2) | 18 (6/6/6) | 6 (2/2/2) | 6 (2/2/2) | 6 (2/2/2) | 18 (6/6/6) |
| *C. utilis* | – | 3 (1/1/1) | 3 (1/1/1) | 6 (2/2/2) | 12 (4/4/4) | 4 (1/2/1) | 6 (2/2/2) | 6 (2/2/2) | 16 (5/6/5) | 4 (1/2/1) | 4 (1/2/1) | 4 (1/2/1) | 12 (3/6/3) |

We expected at maximum 3 and 9 results of correct detections daily or overall, respectively, for mono-species *Candida* cultures, and 6 and 18 results of correct detections daily and overall, respectively, for dual-species *Candida* cultures. In parenthesis, we reported the single detection results by each of the three readers. Dash indicates the result by the reader who judged the growth of *Candida* species on the plate not so enough to allow the discrimination of colonies. Zero indicates the result by the reader who was unable to detect at least one colony due to the absence of growth of *Candida* species on the plate. The media used for the cultures were BCG (Candida bromcresol green), CHROM (chromogenic medium, i.e. Brilliance Candida agar) and SDA (Sabouraud dextrose agar).
