# Supplementary material for: Different detection capabilities by mycological media for Candida isolates from mono- or dual-species cultures
Source: PLoS One. 2020 Mar 23;15(3):e0226467. doi: 10.1371/journal.pone.0226467 (PMC7089522; doi:10.1371/journal.pone.0226467)
Supplement: S2 Table — Bold indicates statistically significant differences in the comparisons between reader #1 and reader #2, reader #2 and reader #3, or reader #1 and reader #3. (DOC) [file pone.0226467.s002.doc]

**S2 Table**

|  | **BCG** | | | **Level of significance (p)** | | | **CHROM** | | | **Level of significance (p)** | | | **SDA** | | | **Level of significance (p)** | | |
| --- | --- | --- | --- | --- | --- | --- | --- | --- | --- | --- | --- | --- | --- | --- | --- | --- | --- | --- |
|  | **Reader #1** | **Reader #2** | **Reader #3** | **Reader #1 vs. Reader #2** | **Reader #2 vs. Reader #3** | **Reader #1 vs. Reader #3** | **Reader #1** | **Reader #2** | **Reader #3** | **Reader #1 vs. Reader #2** | **Reader #2 vs. Reader #3** | **Reader #1 vs. Reader #3** | **Reader #1** | **Reader #2** | **Reader #3** | **Reader #1 vs. Reader #2** | **Reader #2 vs. Reader #3** | **Reader #1 vs. Reader #3** |
| % of incorrect detection at: |  |  |  |  |  |  |  |  |  |  |  |  |  |  |  |  |  |  |
| 24-h reading | 26.9 | 21.1 | 21.1 | 0.20 | 1.00 | 0.20 | 55.6 | 36.8 | 47.4 | **<0.001** | 0.05 | 0.12 | 39.2 | 18.1 | 31.6 | **<0.001** | **0.004** | 0.14 |
| 48-h reading | 15.2 | 13.5 | 13.5 | 0.64 | 1.00 | 0.64 | 35.7 | 17.5 | 18.7 | **<0.001** | 0.77 | **<0.001** | 22.8 | 9.9 | 19.3 | **0.001** | **0.01** | 0.42 |
| 72-h reading | 14.0 | 9.9 | 12.9 | 0.24 | 0.39 | 0.75 | 30.4 | 9.4 | 12.3 | **<0.001** | 0.38 | **<0.001** | 21.1 | 7.6 | 17.5 | **<0.001** | **0.005** | 0.41 |

Mycological media used for mono- or dual-species cultures of *Candida* species were BCG (Candida bromcresol green), CHROM (chromogenic medium, i.e. Brilliance Candida agar) and SDA (Sabouraud dextrose agar).
