# Supplementary material for: Different detection capabilities by mycological media for Candida isolates from mono- or dual-species cultures
Source: PLoS One. 2020 Mar 23;15(3):e0226467. doi: 10.1371/journal.pone.0226467 (PMC7089522; doi:10.1371/journal.pone.0226467)
Supplement: S1 Fig — (DOC) [file pone.0226467.s003.doc]

**S1 Fig**

|  |  | 1 | 2 | 3 | 4 | 5 | 6 | 7 | 8 | 9 | 10 | 11 | 12 | 13 | 14 | 15 | 16 | 17 | 18 |
| --- | --- | --- | --- | --- | --- | --- | --- | --- | --- | --- | --- | --- | --- | --- | --- | --- | --- | --- | --- |
|  |  | **ALB** | **AUR** | **DUB** | **GLA** | **GUI** | **INC** | **KEF** | **KRU** | **LUS** | **NIV** | **NOR** | **PRP** | **PRR** | **PEL** | **ROB** | **SOR** | **TRO** | **UTI** |
| A | **ALB** | A1 | - | - | - | - | - | - | - | - | - | - | - | - | - | - | - | - | - |
| B | **AUR** | B1 | B2 | - | - | - | - | - | - | - | - | - | - | - | - | - | - | - | - |
| C | **DUB** | C1 | C2 | C3 | - | - | - | - | - | - | - | - | - | - | - | - | - | - | - |
| D | **GLA** | D1 | D2 | D3 | D4 | - | - | - | - | - | - | - | - | - | - | - | - | - | - |
| E | **GUI** | E1 | E2 | E3 | E4 | E5 | - | - | - | - | - | - | - | - | - | - | - | - | - |
| F | **INC** | F1 | F2 | F3 | F4 | F5 | F6 | - | - | - | - | - | - | - | - | - | - | - | - |
| G | **KEF** | G1 | G2 | G3 | G4 | G5 | G6 | G7 | - | - | - | - | - | - | - | - | - | - | - |
| H | **KRU** | H1 | H2 | H3 | H4 | H5 | H6 | H7 | H8 | - | - | - | - | - | - | - | - | - | - |
| I | **LUS** | I1 | I2 | I3 | I4 | I5 | I6 | I7 | I8 | I9 | - | - | - | - | - | - | - | - | - |
| L | **NIV** | L1 | L2 | L3 | L4 | L5 | L6 | L7 | L8 | L9 | L10 | - | - | - | - | - | - | - | - |
| M | **NOR** | M1 | M2 | M3 | M4 | M5 | M6 | M7 | M8 | M9 | M10 | M11 | - | - | - | - | - | - | - |
| N | **PRP** | N1 | N2 | N3 | N4 | N5 | N6 | N7 | N8 | N9 | N10 | N11 | N12 | - | - | - | - | - | - |
| O | **PRR** | O1 | O2 | O3 | O4 | O5 | O6 | O7 | O8 | O9 | O10 | O11 | O12 | O13 | - | - | - | - | - |
| P | **PEL** | P1 | P2 | P3 | P4 | P5 | P6 | P7 | P8 | P9 | P10 | P11 | P12 | P13 | P14 | - | - | - | - |
| Q | **ROB** | Q1 | Q2 | Q3 | Q4 | Q5 | Q6 | Q7 | Q8 | Q9 | Q10 | Q11 | Q12 | Q13 | Q14 | Q15 | - | - | - |
| R | **SOR** | R1 | R2 | R3 | R4 | R5 | R6 | R7 | R8 | R9 | R10 | R11 | R12 | R13 | R14 | R15 | R16 | - | - |
| S | **TRO** | S1 | S2 | S3 | S4 | S5 | S6 | S7 | S8 | S9 | S10 | S11 | S12 | S13 | S14 | S15 | S16 | S17 | - |
| T | **UTI** | T1 | T2 | T3 | T4 | T5 | T6 | T7 | T8 | T9 | T10 | T11 | T12 | T13 | T14 | T15 | T16 | T17 | T18 |

The *Candida* species (isolate) used in the study were as follows: ALB, *C. albicans* (UCSC34/23*)*; AUR, *C. auris* (CWZ-1); DUB, *C. dubliniensis* (UCSC35/12); GLA, *C. glabrata* (UCSC61/2); GUI, *C. guilliermondii* (UCSC36/14); INC, *C. incospicua* (UCSC72/2); KEF, *C. kefyr* (UCSC51/14); KRU, *C. krusei* (UCSC59/12); LUS, *C. lusitaniae* (UCSC59/18); NIV, *C. nivariensis* (UCSC11/3); NOR, *C. norvegensis* (UCSC64/13); PRP, *C. parapsilosis* (UCSC30/27); PRR, *C. pararugosa* (UCSC35/20); PEL, *C. pelliculosa* (UCSC72/2); ROB, *C. robusta* (UCSC54/2); SOR, *C. sorbosa* (UCSC28/45); TRO, *C. tropicalis* (UCSC49/29); UTI, *C. utilis* (UCSC36/21).
