# Supplementary figures and images for: Different detection capabilities by mycological media for Candida isolates from mono- or dual-species cultures
Source: PLoS One. 2020 Mar 23;15(3):e0226467. doi: 10.1371/journal.pone.0226467 (PMC7089522; doi:10.1371/journal.pone.0226467)

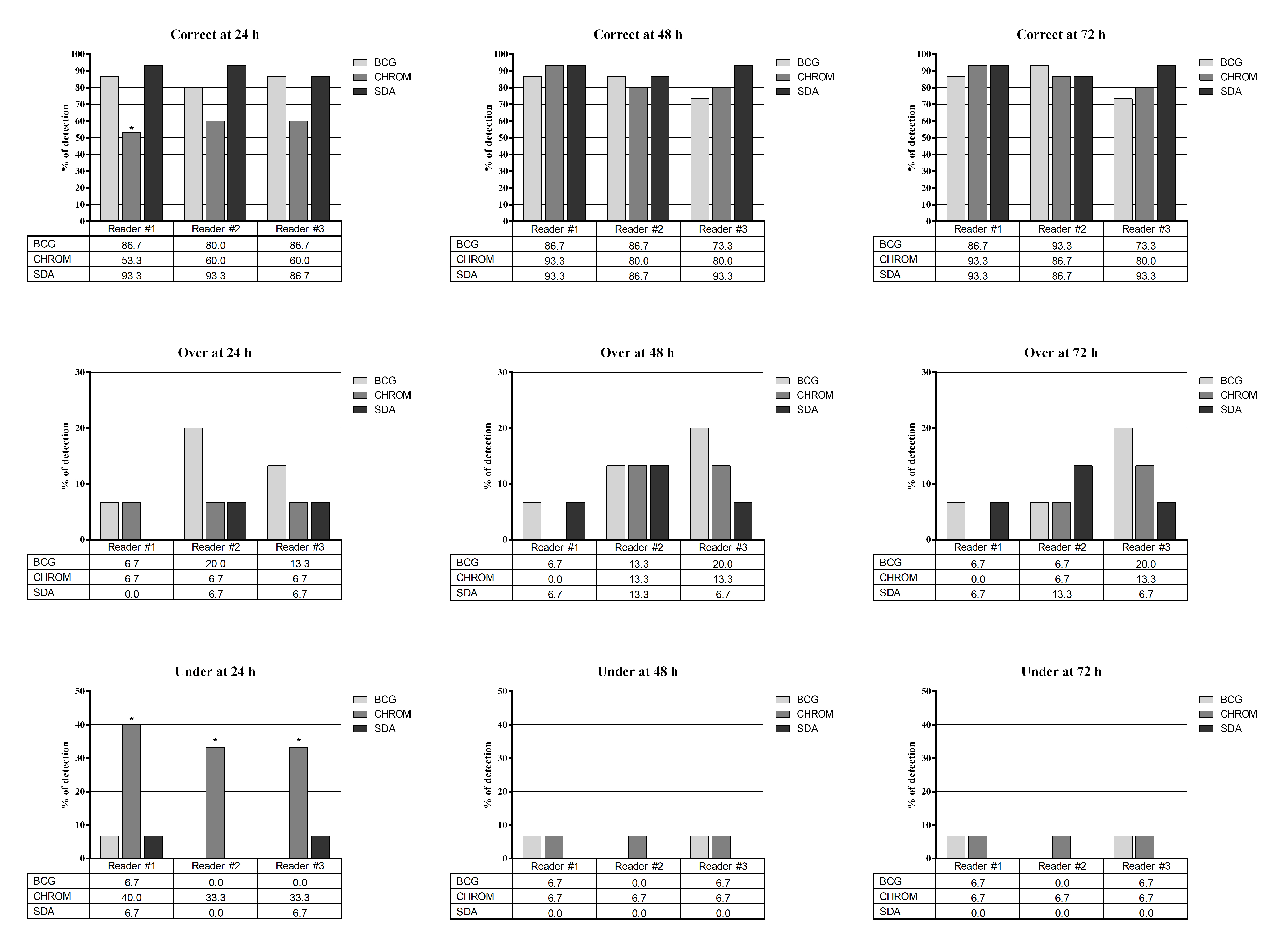

Supplement: S2 Fig — Asterisks indicate statistically significant differences between the rates of detections obtained with the BCG medium and those of the CHROM or SDA media. (JPG) [file pone.0226467.s004.jpg]

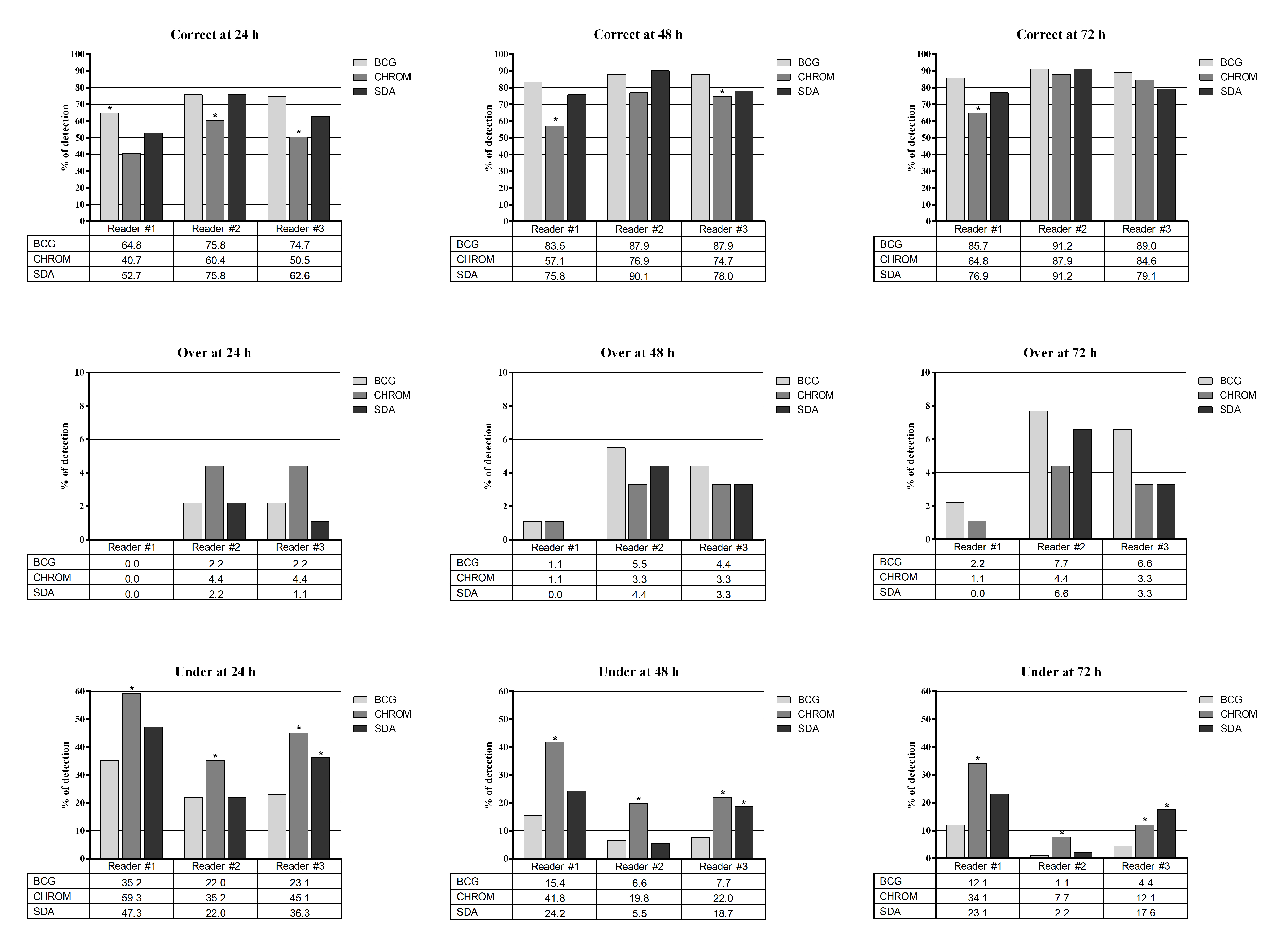

Supplement: S3 Fig — Asterisks indicate statistically significant differences between the rates of detections obtained with the BCG medium and those of the CHROM or SDA media. (JPG) [file pone.0226467.s005.jpg]
