# Supplementary material for: Different detection capabilities by mycological media for Candida isolates from mono- or dual-species cultures
Source: PLoS One. 2020 Mar 23;15(3):e0226467. doi: 10.1371/journal.pone.0226467 (PMC7089522; doi:10.1371/journal.pone.0226467)
Supplement: Attachment — Submitted filename: PONE-D-19-32923 Response to Reviewers.doc [file pone.0226467.s006.doc]

PONE-D-19-32923

1. Please ensure that your manuscript meets PLOS ONE's style requirements, including those for file naming. The PLOS ONE style templates can be found at http://www.journals.plos.org/plosone/s/file?id=wjVg/PLOSOne_formatting_sample_main_body.pdf [http://www.journals.plos.org/plosone/s/file?id=ba62/PLOSOne_formatting_sample_title_authors_affiliations.pdf](https://eur03.safelinks.protection.outlook.com/?url=http%3A%2F%2Fwww.journals.plos.org%2Fplosone%2Fs%2Ffile%3Fid%3Dba62%2FPLOSOne_formatting_sample_title_authors_affiliations.pdf&data=02|01|Brunella.Posteraro@unicatt.it|43ce8a42355c4e491b1208d7992f81b1|b94f7d7481ff44a9b5886682acc85779|0|1|637146301631936453&sdata=sqcZ6UkFjoPoWDcMMmTK01ItZzlIM6CuwBO4p76pFQA%3D&reserved=0)

**Answer: We ensured that our manuscript was in line with the PLOS ONE's style requirements. See the revised manuscript throughout.**

2. For reproducibility purposes, please provide the ID # for all clinical isolates used in this analysis.

**Answer: We provided the ID # for all the clinical isolates used in our analysis. See the text and the footnote of S1 Fig.**

3. Thank you for stating the following in the Acknowledgments Section of your manuscript:

'This study was supported by Università Cattolica del Sacro Cuore (Fondi Ateneo, linea D1-2018).'

We note that you have provided funding information that is not currently declared in your Funding Statement. However, funding information should not appear in the Acknowledgments section or other areas of your manuscript. We will only publish funding information present in the Funding Statement section of the online submission form.

Please remove any funding-related text from the manuscript and let us know how you would like to update your Funding Statement. Currently, your Funding Statement reads as follows:

'The author(s) received no specific funding for this work.'

**Answer: We apologize for the incongruence of our statements. You will find the updated statements in the Funding Section.**

Reviewer #1: In this work, the Authors compared the performance of 3 different mycological media, namely BCG, CHROM and the non-chromogenic SDA, for the detection of different Candida species cultures, both in mono-species and dual-species culture plates. Plates were read at 24, 48 and 72 hrs from 3 independent readers, and results were interpreted as correct detection, over detection, or under detection. Overall, BCG medium showed the best performance with higher rates of correct detection and lower rates of under detection for all Candida species analyzed, but also by splitting Candida spp. in common and uncommon species, although with differences in the statistical significance. The Authors conclude BCG medium may represent an accurate tool for the correct identification to the species level and for the laboratory diagnosis of candidemia.

The matter of the manuscript is of interest in clinical diagnostics and provides valuable indications for the microbiology laboratory in the accurate diagnosis of Candida infections.

Some considerations:

- From what stated in the text, it would be interesting to know the opinion for the right time and temperature of incubation of plates for clinical specimens for the isolation of Candida spp, also as indicated by the literature.

**Answer: We provided details about the optimal time and temperature of incubation of plates for clinical specimens that are required for the isolation of *Candida* species. See lines 100–102 of the revised manuscript.**

- It is not clear if the term “over detection” means culture with more colony morphotypes, but not necessarily different species, or more Candida species than those expected. Please, clarify it.

**Answer: We clarified this issue adding a sentence as appropriate. See lines 111–113 of the revised manuscript.**

- It would be interesting to know the true clinical impact of mixed Candida infections, specifically in candidemia (page 8, lines 170-174, the mixed cultures reported seems to be very uncommon, right?).

**Answer: We added a comment about this interesting aspect raised by the reviewer. See lines 199–201 of the revised manuscript.**

- page 5, line 84-88. Please, it is necessary to better specify the final volume (the final concentration) reached in each well. Could the Authors provide some references to support their choice for the methodologies?

**Answer: We specified the final volume reached in each well as well as provided a reference to support our methodological choice. See lines 88–92 of the revised manuscript.**

- Page 8, lines 161-165. I would add this part also in the Results section along with the reference of Fig 4, with a brief description of the characteristics of the Candida media used.

**Answer: We added the part indicated by the reviewer in the Results section along with the reference of Fig 2 (formerly Fig 4) and a brief description of the media’s characteristics. See lines 155–164 of the revised manuscript.**

- The resolution of Figures 1, 2, 3 needs to be much improved, they are blurred. Moreover, it is also difficult to notice what the Authors state for the Figure 4 with regard to the differences in the colony morphologies.

**Answer: We improved all the Figures, namely Fig 1, S1 Fig (formerly Fig 2) and S2 Fig (formerly Fig 3) for quality. In particular, we redone the image of Fig 2 (formerly Fig 4) to make more evident the differences in the colony morphologies of the isolates photographed.**

- There is a variability between reader 1 and the others respect to the over detection at 24, 48 and 72 hrs mainly for all Candida species and the uncommon Candida species, as indicated in the Figures 1 to 3 and in Table 1 (moderate or fair levels of agreement).What is the reason of it? Does this could affect the results?

**Answer: We added a comment about this interesting aspect raised by the reviewer. See lines 185–189 of the revised manuscript.**

Reviewer #2: The ms by De Angelis et al is a comparison study among different commercially available media (three) in order to understand if these media can be used for detecting different Candida spp from primary cultures.

The AA used 18 strains belonging to different species of Candida, all isolates from BSI. A C. auris strain was also included. Following a CK scheme, cultures were variably combined to obtain mono, dual and triple combination of strains. All these unique combinations were plated on the three media and results were reported in 1 table and many figures.

The ms is clearly written and results obtained, even with limitations described by the AA (reading plates remain very subjective, and the different morphotype of Candida) are in favor of the use of BCG.

In my opinion, two minor points need to be addressed:

1) The measure of the level of inter reader agreement is somewhat vague. Any comment from the AA?

Is it possible to measure this agreement in term of errors?

**Answer: We agree with the reviewer on the vagueness of measuring the level of inter-reader agreement. Unfortunately, we were unable to measure the agreement in terms of errors. However, we added data about the rates of incorrect (over/under) detections of the three readers in order to underline the statistically significant differences between the readers with respect to the incorrect reading results. See lines 151–154 and a new Table in the Supporting information (S2 Table) of the revised manuscript.**

2) Number of figures can be reduced, summarizing the most significative results without losing the meaning of the message (the original figures can be added at the supplemental material section).

**Answer: As suggested, we move Fig 2 (now S1 Fig) and Fig 3 (now S2 Fig) to Supporting information.**
